# High Gamma Oscillations in Medial Temporal Lobe during Overt Production of Speech and Gestures

**DOI:** 10.1371/journal.pone.0111473

**Published:** 2014-10-23

**Authors:** Lars Marstaller, Hana Burianová, Paul F. Sowman

**Affiliations:** 1 Centre for Advanced Imaging, University of Queensland, Brisbane, Australia; 2 ARC Science of Learning Research Centre, University of Queensland, Brisbane, Australia; 3 ARC Centre of Excellence in Cognition and its Disorders, Macquarie University, Sydney, Australia; 4 Department of Cognitive Science, Macquarie University, Sydney, Australia; Hospital for Sick Children, Canada

## Abstract

The study of the production of co-speech gestures (CSGs), i.e., meaningful hand movements that often accompany speech during everyday discourse, provides an important opportunity to investigate the integration of language, action, and memory because of the semantic overlap between gesture movements and speech content. Behavioral studies of CSGs and speech suggest that they have a common base in memory and predict that overt production of both speech and CSGs would be preceded by neural activity related to memory processes. However, to date the neural correlates and timing of CSG production are still largely unknown. In the current study, we addressed these questions with magnetoencephalography and a semantic association paradigm in which participants overtly produced speech or gesture responses that were either meaningfully related to a stimulus or not. Using spectral and beamforming analyses to investigate the neural activity preceding the responses, we found a desynchronization in the beta band (15–25 Hz), which originated 900 ms prior to the onset of speech and was localized to motor and somatosensory regions in the cortex and cerebellum, as well as right inferior frontal gyrus. Beta desynchronization is often seen as an indicator of motor processing and thus reflects motor activity related to the hand movements that gestures add to speech. Furthermore, our results show oscillations in the high gamma band (50–90 Hz), which originated 400 ms prior to speech onset and were localized to the left medial temporal lobe. High gamma oscillations have previously been found to be involved in memory processes and we thus interpret them to be related to contextual association of semantic information in memory. The results of our study show that high gamma oscillations in medial temporal cortex play an important role in the binding of information in human memory during speech and CSG production.

## Introduction

Humans routinely produce communicative hand gestures in conjunction with spoken language, i.e., co-speech gestures (CSGs). In half of all CSGs, the hand movements express the spoken language's verbal meaning in visuo-spatial form, e.g., when the utterance “and then the airplane took off like this” is accompanied by the speaker's flat hand moving forward and upwards [1], [2]. This semantic combination of speech and hand movements makes CSGs a unique phenomenon for the study of the relationship between language, action, and memory in the human brain.

The extensive overlap of the meaning of a hand gesture with the semantic content of concurrent speech suggests that CSGs combine different aspects of memory into a single multimodal expression. During the production of CSG memory processes relating to action, visuo-spatial cognition, and language are combined into a meaningful whole, in which the meaning of speech content and gesture movements support each other. The semantic integration of speech and CSGs has led researchers to hypothesize that speech and CSGs might share a common base in memory [2]–[4], and thus mainly interact during early, memory-related stages of speech/gesture production. Given that speech and CSGs engage different memory representations, this interaction should be reflected in coordinated neural activity relating to semantic processes of verbal-linguistic (for speech) and visuo-spatial (for CSGs) content. However, to date the neural correlates of the memory and motor planning processes underlying the production of CSGs are largely unknown. We address this issue in this study, using magnetoencephalography (MEG) to measure neural activity prior to overt production of speech and CSGs in a semantic association task. MEG is optimal for delineating timing-dependent neural correlates, as it combines high temporal resolution, allowing for the investigation of the neural activity preceding the onset of speech and CSGs, with the ability to spatially localize functional activity in the brain.

MEG has been used previously to investigate the neural correlates of human memory. Studies investigating long-term memory have found neural activity in medial temporal, frontal, and posterior parietal regions for episodic or recognition memory [5]–[8], and semantic association memory [9]–[11]. It has been suggested that memory encoding, maintenance, and retrieval are the result of an interaction between fast rhythms in local neural populations, which form functionally distinct areas, and slow rhythms, which integrate neural activity across brain regions [12]–[17]. As speech and CSGs engage different memory representations (verbal-linguistic for speech and visuo-spatial for gesture movements), it is reasonable to predict that neural activity at higher frequencies would relate to functionally distinct memory processes preceding the overt production of speech or CSGs, whereas neural activity in lower frequencies would be indicative of integrative processes related to both speech and CSGs. Research on speech production suggests that these memory processes would involve temporal regions in the left hemisphere responsible for semantic association and that these memory systems are distinct from procedural systems responsible for the production of action sequences [18]–[20]. Previous studies using MEG to investigate language production found that activity in prefrontal cortex at frequencies between 15 and 35 Hz is involved in language production [21], [22]. In addition, electrocorticography studies found that language processes engage high gamma band activity (50–200 Hz) in temporal regions [23], [24].

The goal of this study was to use MEG to examine the time-frequency spectrum for differences and commonalities in the neural correlates of memory processes, which are related to the retrieval and contextualization of semantic content at the early stages of speech and CSG. Based on previous studies [7], [25], we hypothesized that we would find oscillations in the gamma band in sensory and higher association areas that correlate with retrieval and processing of verbal-linguistic and visuo-spatial content for speech or CSG. We further hypothesized that we would find oscillations in the theta band, which are associated with the large-scale integration of information across brain regions during both speech and CSG. In addition to memory-related oscillatory changes, we also expected to find evidence in the beta band of the motor and somatosensory cortex relating to the increased demands of planning and executing hand movements that have to be added to speech during CSG production [26].

## Methods

### Participants

16 right-handed, healthy participants (mean age  = 29 years; range  = 22–37 years; 9 females), with normal or corrected to normal vision, took part in the experiment. All participants acquired English as a primary language before the age of four years and received 12 or more years of formal education. The human research ethics committee of Macquarie University approved this study and written consent was obtained from all participants.

### Stimulus Set and Experimental Design

The stimulus set contained 90 nouns, which referred to common objects that can be manipulated by hand, as well as the nonsensical character string “#%$&@” as a control stimulus. The association task required the participants to overtly respond to a visually presented stimulus. Stimuli and responses differed along two dimensions. First, we manipulated whether the stimulus had a meaning by presenting either a noun (meaningful) or the control stimulus (meaningless). Second, we instructed the participants to respond through speech, gesture, or the combination of speech and gesture (CSG). For the semantically related, meaningful stimuli, participants were asked to either produce a verb or a hand gesture that was associated with the presented noun or both in conjunction. For the semantically unrelated control stimuli, participants were asked to either overtly produce the nonsense syllable/*ga*/, a pinching gesture without specific meaning, or both the nonsense syllable and the pinching gesture in conjunction.

For each response, speech onset was detected with a microphone that was mounted in the magnetically shielded room and connected to a computer outside of the magnetically shielded room, which ran Presentation software (Neurobehavioral Systems, Inc.). Due to technical reasons, the onsets of the gesture hand movements were not recorded and responses requiring hand movements without speech are thus excluded from the analysis. Special care was taken to ensure that participants understood that their gestures could cause head movements and they were thus explicitly instructed to only move their right lower arm and hand, and gesture with small, short, and smooth movements. Prior to the experiment, participants practiced the task for approximately 10 minutes, using stimuli different from those used in the experiment.

**Figure 1:**
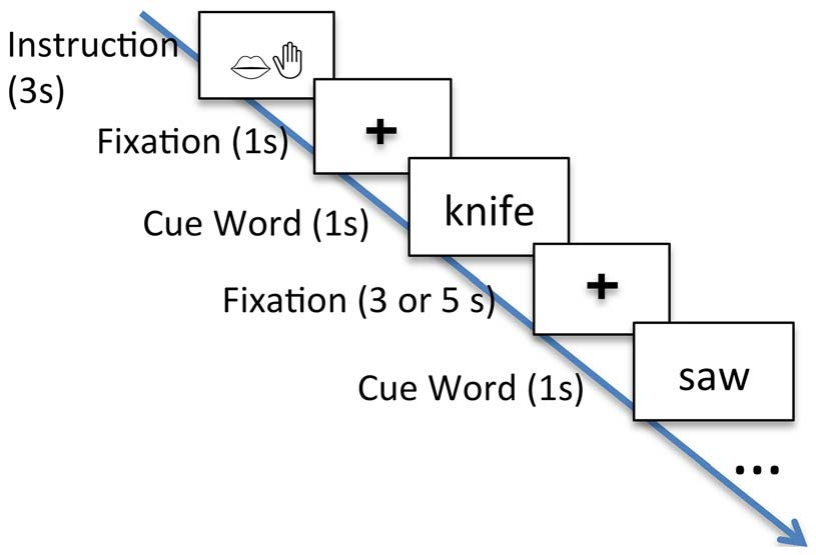
Task. After an instruction, participants are presented with a number of meaningful or control stimuli and produce either a meaningful response or a predefined response to each stimulus. In addition, responses are either unimodal (speech) or bimodal (speech and gesture, i.e., co-speech gesture).

In the meaningful conditions, each noun was presented once per response type, i.e., each noun was seen three times by each participant. A control for standard psycholinguistic variables, such as age of acquisition, frequency, length, or neighborhood size was not employed because any potential impact would affect each response type equally. Stimuli were presented in blocks of 10 items in randomized order, resulting in nine blocks per condition. Each block started with the presentation of an instruction, such as ‘Produce words’ or ‘Produce words and gesture’ for 3 sec. In each meaningful trial, the stimulus was presented for 1 sec followed by a fixation cross for 5 sec. In each control trial, the stimulus was presented for 1 sec followed by a fixation cross for 3 sec (see Fig. 1). The order of conditions was randomized within blocks of meaningful and control trials, and the order of blocks of meaningful and control trials was counterbalanced across individuals.

### MEG Data Acquisition and Preprocessing

Prior to MEG recordings, marker coil positions and head shape were measured with a pen digitizer (Polhemus Fastrack, Colchester, VT). MEG recordings were obtained from participants in a supine position in a magnetically shielded room (Fujihara Co. Ltd., Tokyo, Japan) using the KIT-Macquarie MEG160 (Model PQ1160R-N2, KIT, Kanazawa, Japan). Data were recorded using 160 coaxial first-order gradiometers with a 50 mm baseline [27], [28]. MEG data were acquired with a sampling rate of 1000 Hz and a bandpass filter of 0.03–200 Hz. All subsequent offline data processing was performed with Statistical Parametric Mapping software for M/EEG (SPM 8; http://www.fil.ion.ucl.ac.uk/spm). Data were downsampled to 250 Hz prior to analysis. To eliminate low frequency and electrical noise, a bandpass filter with cut-off of 0.1 and 100 Hz and a stop band filter ranging from 49 to 51 Hz were applied. Data were epoched from −2100 to 1600 ms relative to the speech onset. Artifacts due to blinks, jaw or eye-movements, were removed for each trial using visual artifact rejection implemented in SPM8 [29].

### Time-Frequency Analysis

Time-frequency analysis was conducted on the signal averaged over all channels in the frequency range between 0.1 and 100 Hz. Power was analyzed in 0.5 Hz steps using Morlet wavelets with a seven-cycle width [30]. Epochs were averaged within conditions and the resulting average epoch was cropped from −2000 to 1500 ms to remove edge effects. The resulting spectra were then rescaled to a baseline time-window which we defined as the epoch from −2000 to −1500 ms. To assess statistically significant differences in the spectral profiles, the individual spectrograms were converted to statistical parametric images and entered into a 2×2 random effects analysis of variance with the factors response (unimodal speech and bimodal CSG) and condition (meaningful and control). To correct for multiple comparisons, a family-wise error (FWE) correction using Gaussian random field theory method was employed [31] and resulting statistical parametric maps were thresholded at p<0.05. Additional t-tests that were conducted to investigate the differences between conditions of interest were also thresholded at p<0.05.

### Beamformer Source Localization

To localize the spatial origin of the neural signals found in the time-frequency analysis, two separate beamformer analyses were conducted. For the first analysis a time window ranging from 200 to 600 ms relative to speech onset in the beta frequency band (15–25 Hz) was chosen because it reflected statistical significant differences between CSG and speech responses in the time-frequency analysis. For the second analysis, a time window from −100 to 100 ms relative to speech onset in the gamma frequency band (50–90 Hz) was selected because of its statistical significance for meaningful as compared to meaningless trials in the time-frequency analysis. For both analyses, a linearly constrained minimum variance (LCMV) beamforming algorithm implemented in SPM8 with a 5 mm grid size and 1% regularization was used. This method imposes eigenvalues of the covariance matrix as linear constraints on the minimization of the weights of a spatial filter [32]. For both analyses, the individual results were separately entered into a random effects 2×2 ANOVA with the same factors as in the time-frequency analysis, i.e., response (unimodal speech and bimodal CSG) and condition (meaningful and control). For the source maps returned for each time-frequency window, F-contrasts and t-contrasts for each of the four experimental conditions were calculated. Finally, to correct for multiple comparisons, a family-wise error correction was applied and statistical maps were thresholded at p<0.0001. For technical reasons, one dataset was excluded from the beamformer analysis.

## Results

### Behavioral Performance

To assess the speed of speech production, latencies between the onset of the cue word and the verbal response were calculated in the speech and CSG conditions. Speech onsets were defined as the first verbal response (voice-key trigger) following the stimulus presentation. Average response times and standard deviations were first computed for each individual and trial type and then averaged across the group. In the meaningful trials, participants were significantly faster to produce verbs in the speech condition (mean latency  = 1396 ms, SD  = 207 ms) than in the CSG condition (mean latency  = 1634 ms, SD  = 319 ms); t(14)  = 6.668, p<0.001. This difference is interesting but does not affect analysis of the MEG data because it is related to neural activity preceding the speech onset and should therefore not be confounded by differences in production speed. Participants were also faster in producing verbal responses in the control trials but there was no significant difference between the control speech condition (mean latency  = 857 ms, SD  = 434 ms) and the control CSG condition (mean latency  = 941 ms, SD  = 406 ms); t(14)  = 1.331, p = 0.205.

### Time-Frequency Analysis

**Figure 2:**
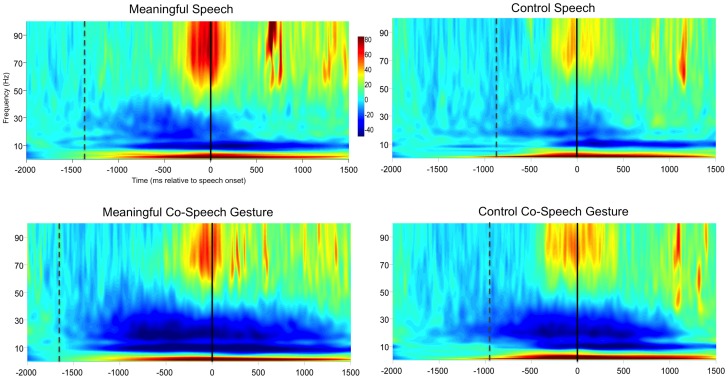
Results of time-frequency analysis for meaningful speech, meaningful co-speech gesture, control speech, and control co-speech gesture. Statistical analysis shows (i) a main effect of response, i.e., stronger beta-desynchronization for co-speech gesture than speech responses, and (ii) a main effect of condition, i.e., stronger gamma oscillations for meaningful than control trials. Solid lines indicate speech onset and dashed lines average time of stimulus presentation.

**Figure 3:**
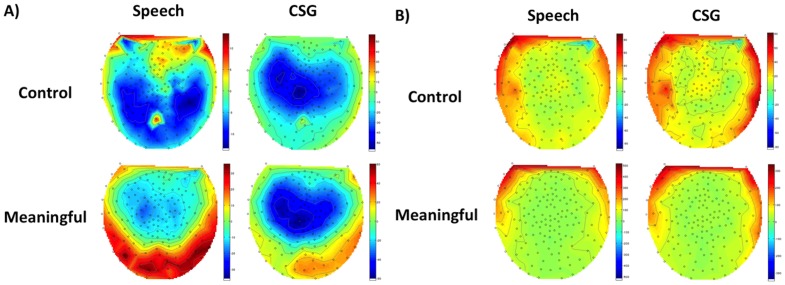
Topographical maps of the time-frequency responses for meaningful and control trials. Dashed lines show the average onset time of speech responses for the beta time-frequency window (15–25 Hz, 200 to 600 ms post speech onset) (A) and the high gamma time-frequency window (50–90 Hz, –100 to 100 ms post speech onset) (B).

ANOVA of the time frequency plots showed a significant main effect of response in the beta band (15–25 Hz) between approximately −750 ms and 950 ms relative to speech onset (F(1,56)  = 21.6, p<0.05 FWE), revealing a stronger beta event-related desynchronization in bimodal CSG responses than in unimodal speech responses (t(56) = 4.42, p<0.05 FWE). In addition, the results of the ANOVA show a significant main effect of condition in the gamma band (45–100 Hz) between approximately −425 ms and 100 ms relative to speech onset (F(1,56)  = 21.6, p<0.05 FWE), displaying stronger high gamma oscillations during responses to meaningful nouns than the control string (t(56) = 4.42, p<0.05 FWE). Even though we found no significant interaction between response and condition, visual inspection of the time frequency plots suggests earlier, stronger, and more sustained high gamma oscillations in the speech than in the CSG condition (see Fig. 2 & 3).

### Beamformer Analysis

**Figure 4:**
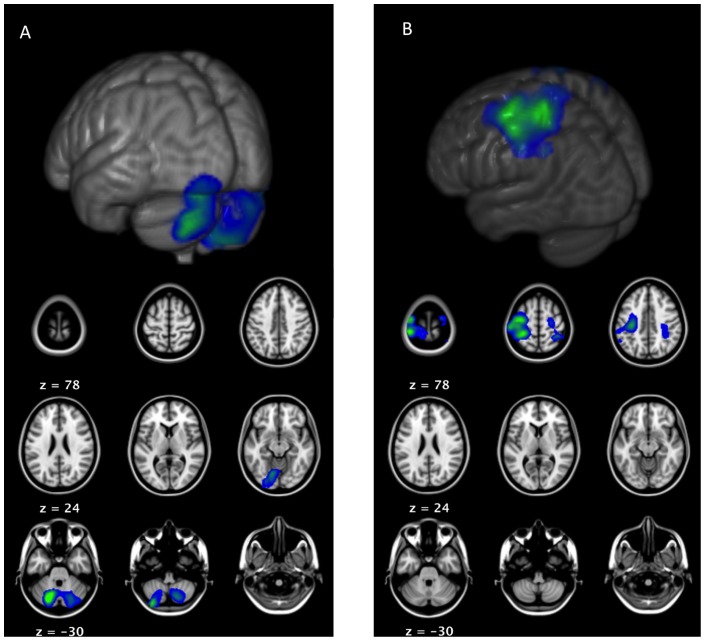
Results of beamformer analysis for beta band time frequency window (15–25 Hz, 200 to 600 ms post speech onset). The ANOVA of the source images shows (i) a main effect of condition localized to bilateral cerebellum, right fusiform and lingual gyri, and posterior cingulate cortex related to meaningful trials (A); and (ii) a main effect of response localized to bilateral motor and somatosensory areas and related to the additional motor control required for hand movement during the co-speech gesture responses (B).

Beamformer analysis was conducted on two statistically significant time-frequency windows: one in the beta band differentiating between speech and CSG responses (15–25 Hz, 200 to 600 ms) and one in the high gamma band distinguishing between meaningful and control trials (50–90 Hz, −100 to 100 ms). A 2×2 ANOVA of the beta band time-frequency window shows a main effect of response localized to bilateral primary somatosensory and motor cortices, and a main effect of condition localized to bilateral cerebellum (both F(1,52)  = 23.5, p<0.05 FWE; see Fig. 4). The activity in somatosensory and motor cortices is related to the CSG response as compared to speech (t(52)  = 4.61, p<0.05 FWE). In addition, t-tests comparing meaningful and control conditions show that activity in the cerebellum is related to the control conditions and activity in right inferior frontal gyrus is related to the semantic conditions (both t(52)  = 4.61, p<0.05 FWE). These results show functional beta desynchronization in two separate brain regions, first, in motor and somatosensory cortices as well as the cerebellum related to hand movements and second, in the right inferior frontal gyrus related to semantic processing.

**Figure 5:**
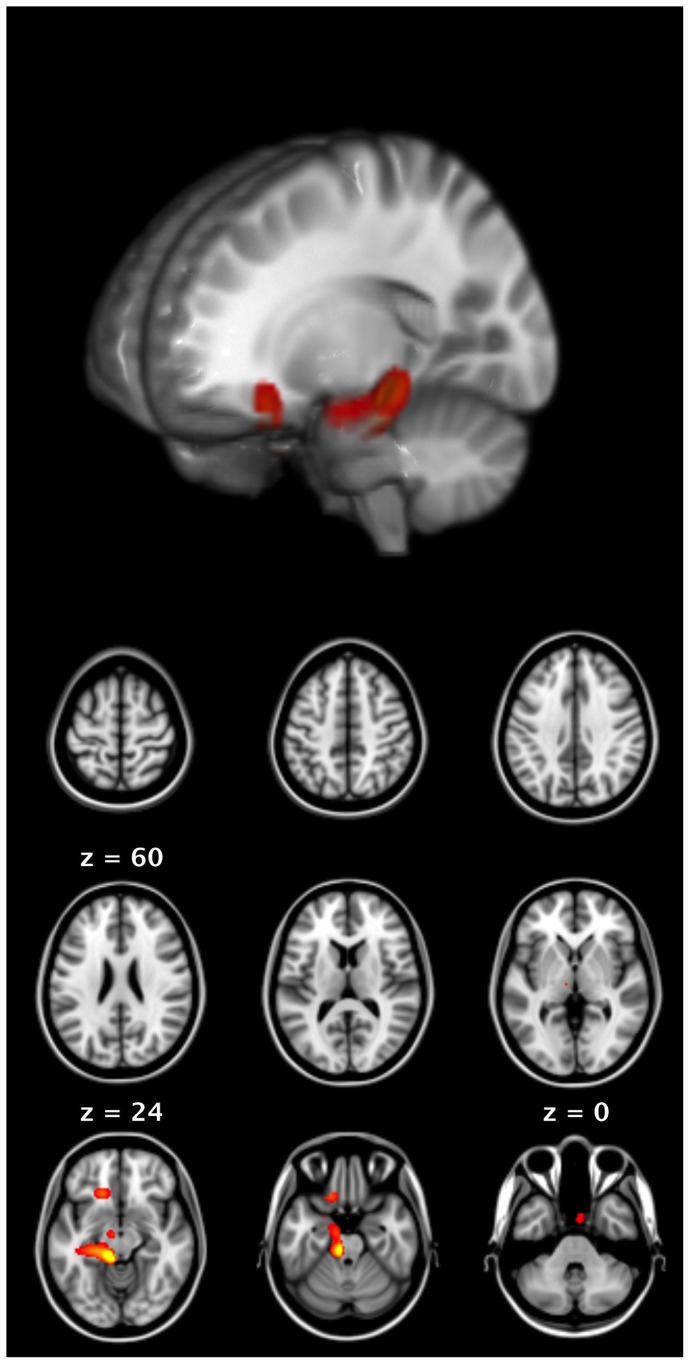
Results of beamformer analysis for gamma band time frequency window (50–90 Hz, −100 to 100 ms relative to speech onset). The ANOVA of the source images shows a main effect of condition localized to left medial temporal lobe, striatum, thalamus, and ventromedial prefrontal cortex related to meaningful trials.

A 2×2 ANOVA of the high gamma band time-frequency window shows a main effect of condition localized to left medial and lateral temporal lobe (MTL), striatum, thalamus, right cerebellum, and bilateral ventral medial prefrontal cortex (F(1,52)  = 21.68, p<0.05 FWE; see Fig. 5). T-tests show that this effect is due to meaningful trials only (t(52)  = 4.41, p<0.05 FWE). Additional t-tests, comparing meaningful and control trials within unimodal and bimodal responses, show stronger high gamma oscillations in medial temporal regions for speech than for CSG (t(52)  = 4.41, p<0.05 FWE).

**Figure 6:**
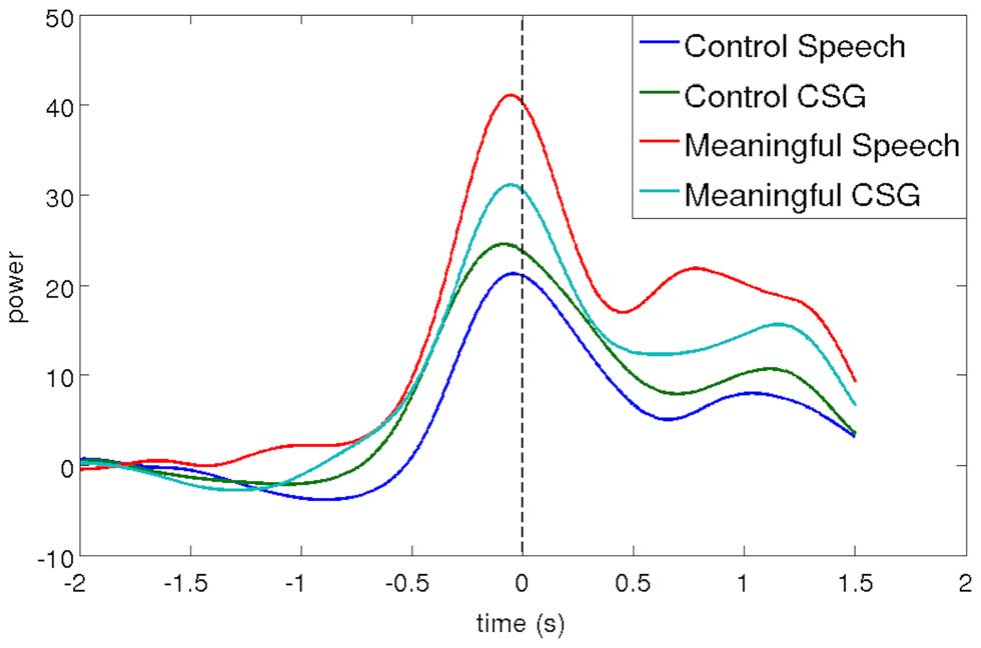
Smoothed power envelopes of high gamma responses extracted from left medial temporal lobe using beamforming. The different curves show a similar pattern for speech and CSG in control trials but a different pattern for speech and CSG in meaningful trials.

High gamma oscillations might be related to cognitive processes but they could also reflect muscle movements [33]. To further investigate the source of the observed high gamma oscillations, we used a spatial beamforming filter to extract the time-frequency response in the left medial temporal lobe at MNI coordinates −29 −29 −11 (see Fig. 6). The figure shows stronger power increases for the experimental than the control conditions, which is in accordance with the experimental manipulation and confirms the previous t-tests. In addition, the figure shows no difference between the two control conditions but suggests a different pattern for the experimental conditions, where speech production is accompanied by stronger gamma oscillations in comparison to CSG production. If the source of the observed high gamma oscillations was muscle movement, there should be no difference between the two experimental conditions because both involve comparable amounts of overt speech production (as seen in the control conditions). This comparison suggests that the observed gamma oscillations are not due to muscle movement. Taken together these results show high gamma oscillations related to memory processes in subcortical and medial structures of frontal and temporal lobes during speech and CSG production.

## Discussion

The aim of this study was to investigate the neural processes preceding the overt production of speech and co-speech gestures (CSG) and particularly to examine the frequency spectrum for neural activity relating to differences and commonalities in memory retrieval for speech and CSGs. The results are twofold and show first, beta desynchronization in the motor and somatosensory areas in trials that involve hand movements, and in the inferior frontal gyrus only in trials that involve semantic association. The results further show high gamma oscillations in lateral and medial temporal lobes (MTL), as well as in subcortical and medial frontal regions. The first finding reflects motor planning and suggests an engagement of the right inferior frontal gyrus in semantic association of a response with its stimulus. The second finding provides evidence for a contextual binding in associative memory that is common to speech and CSG. Our data do not provide evidence for significant differences between speech and CSG, and hence suggest that speech and CSG might have a common base in memory.

### Beta-Band Desynchronization

We present evidence for neural activity related to planning and execution of overt hand movements by showing beta desynchronization in motor and somatosensory regions that is significantly stronger for responses involving CSG than speech. Beta desynchronization in motor and somatosensory cortex has previously been shown to be related to hand and arm movements [26], [34], [35]. However, while beta desynchronization in motor and somatosensory cortex can be attributed to hand movements, beta desynchronization in the cerebellum was only found for control trials, i.e., for simple repetitive speech and CSG responses but not their meaningful counterparts, which involved semantic processing. We suggest that this finding is indicative of motor control and procedural memory processes in the cerebellum [36]. In addition, our results also show that beta desynchronization in the right inferior frontal gyrus (IFG) related only to meaningful experimental but not the control trials. Left IFG has been reported to be involved in speech production, specifically during motor planning and response sequencing [20], and responses in experimental trials, where participants produced whole words, are more complex than in control trials, where participants produced a single syllable. We therefore suggest that beta ERD in right IFG reflects the increased demands for motor planning in experimental trials.

### High-Gamma Oscillations

Our results show high gamma oscillations in MTL for meaningful trials, which we propose to be related to associative memory processes because only in these trials do participants search for a meaningful response that is semantically related to a common noun. Other trials, in which participants produce the same response that has no established meaning to the same non-semantic stimulus, show significantly less high gamma oscillations in MTL. Therefore, only meaningful trials, which show high gamma oscillations in MTL, require the retrieval and binding of new content from memory. Studies using functional magnetic resonance imaging or lesion studies show that MTL is involved in long-term memory retrieval [37], [38], encoding of relations in working memory [39]–[41], and resolution of interference related to short-term memory [42]–[44]. Evidence from several studies further suggests that gamma oscillations in MTL are directly related to memory processes, such as memory encoding and maintenance [45], [46], semantic processing [9], picture naming [24], and verb generation [23]. Converging evidence suggests that the function of gamma oscillations in MTL consists of providing contextual associations by binding together information from different cortical sources, such that previously experienced content can be remembered [15], [47]–[49]. Our results also show additional regions, which reflect high gamma oscillations during speech and CSG production, specifically ventromedial prefrontal gyrus, left thalamus, and posterior lateral middle temporal gyrus. These brain areas have been identified as critical nodes underlying semantic and episodic memory processes [50]–[54]. Because our results show high gamma oscillations only for meaningful trials, i.e., those trials that engage semantic association and memory retrieval, we suggest that high gamma oscillations in MTL relate to retrieval of semantic associations from memory during overt production of both speech and CSG.

Furthermore, the timing of memory-related high gamma oscillations in MTL has important implications for models of speech and CSG production. Our results show that gamma oscillations in MTL precede speech onset by up to 450 ms and continue until after speech onset. This timing coincides with a meta-analysis of imaging studies, which found that semantic and lexical processes precede the speech onset in a time window ranging from around −400 to −250 ms [55]. Our results suggest that during this time, high gamma oscillations in MTL perform the function of binding information from different cortical sources together so that memory content (i.e., lexico-semantic items) is available for further processing [15], [49]. The finding that speech and CSGs engage common neural mechanisms related to memory retrieval suggests a potential time window of interaction between different memory systems related to language and action and provides evidence for a common origin of speech and CSG in memory [2]–[4]. In this context, it is interesting to note that our results tentatively show differences in the strength and extent of high gamma oscillations in MTL between speech and CSG, which would be relevant for potential explanations of the behaviorally observed enhancement effect of CSGs on working memory [56], [57]. We realize that due to the somewhat artificial nature of neuroimaging experiments, our results cannot be readily extended without further evidence from naturally occurring CSGs or other types of CSGs beyond iconic CSGs. However, our findings provide important first insight into the neural processes underlying CSG production, showing that semantic association and motor planning are at the core of CSG production, which might be further modulated by other processes related to attention, language, and social cognition.

### Limitations

CSG production is extremely difficult to simulate under experimental conditions, especially in neuroimaging experiments. The responses in our paradigm therefore differ from naturally produced CSGs in important ways. In our task, participants are asked to produce a single gesture in relation to each stimulus noun. In contrast, during naturally occurring discourse, CSGs are produced spontaneously, in relation to a whole spoken clause, and with reference to the discourse context (McNeill, 1992). In addition, there are different types of naturally produced CSGs, which differ in important aspects of timing, semantics, and relation between speech and gesture, whereas in our task, participants produced only one type of CSGs, so called iconic CSGs, which visually imitate the actions they refer to [1]. Together, these differences limit the ecological validity of the CSGs we study in our experiment. However, we believe that the CSGs produced in our task share important features with naturally occurring CSGs, such as semantic retrieval based on a linguistic context, control of the appropriateness of the response and of semantic overlap between speech and gesture, as well as motor planning, execution, and monitoring. We further believe that CSGs produced in our task mainly differ with respect to discourse sensitivity from naturally occurring CSGs. As such, we are certain that our paradigm elicits important aspects of the production process of iconic CSGs.

## Conclusions

In sum, our results show that high gamma oscillations in medial temporal lobe are engaged by both speech and CSG. These findings suggest that the production of speech and CSGs both engage binding of contextual information during memory retrieval and semantic association. Our results highlight the role of high gamma oscillations in MTL in the production of speech and CSG as well as the interaction between memory, language, and action.

## References

[1] Kendon A (2004) Gesture: Visible action as utterance. Cambridge: Cambridge University Press.

[2] McNeill D (1992) Hand and mind: what gestures reveal about thought. Chicago, IL: Chicago University Press.

[3] De Ruiter JP (2000) The production of gesture and speech. In: McNeill D, editor. Language and Gesture. Cambridge: Cambridge University Press. 248–311.

[4] Kita S, Özyürek A (2003) What does cross-linguistic variation in semantic coordination of speech and gesture reveal? Evidence for an interface representation of spatial thinking and speaking. Journal of Memory and Language 48: 16–32.

[5] Düzel E, Habib R, Rotte M, Guderian S, Tulving E, et al (2003) Human hippocampal and parahippocampal activity during visual associative recognition memory for spatial and nonspatial stimulus configurations. J Neurosci 23(28): 9439–44. 14561873 10.1523/JNEUROSCI.23-28-09439.2003 PMC6740564

[6] Martin T, McDaniel MA, Guynn MJ, Houck JM, Woodruff CC, et al (2007) Brain regions and their dynamics in prospective memory retrieval: a MEG study. Int J Psychophysiology 64(3): 247–58. 10.1016/j.ijpsycho.2006.09.010 17126436

[7] Osipova D, Takashima A, Oostenveld R, Fernández G, Maris E, et al (2006) Theta and gamma oscillations predict encoding and retrieval of declarative memory. J Neurosci 26(28): 7523–31. 16837600 10.1523/JNEUROSCI.1948-06.2006 PMC6674196

[8] Zion-Golumbic E, Kutas M, Bentin S (2010) Neural dynamics associated with semantic and episodic memory for faces: evidence from multiple frequency bands. J Cogn Neurosci 22(2): 263–77. 19400676 10.1162/jocn.2009.21251 PMC2807899

[9] Mellem MS, Friedman RB, Medvedev AV (2013) Gamma- and theta-band synchronization during semantic priming reflect local and long-range lexical-semantic networks. Brain Lang 127(3): 440–51. 24135132 10.1016/j.bandl.2013.09.003 PMC3864756

[10] Nieuwenhuis IL, Takashima A, Oostenveld R, McNaughton BL, Fernández G, et al (2012) The neocortical network representing associative memory reorganizes with time in a process engaging the anterior temporal lobe. Cereb Cortex 22(11): 2622–33. 22139815 10.1093/cercor/bhr338 PMC3464414

[11] Serruya MD, Sederberg PB, Kahana MJ (2012) Power shifts track serial position and modulate encoding in human episodic memory. Cereb Cortex. [Epub ahead of print]. 10.1093/cercor/bhs318 PMC3888367 23081881

[12] Dipoppa M, Gutkin BS (2013) Flexible frequency control of cortical oscillations enables computations required for working memory. Proc Natl Acad Sci U S A 110(31): 12828–33. 23858465 10.1073/pnas.1303270110 PMC3732977

[13] Jensen O (2006) Maintenance of multiple working memory items by temporal segmentation. Neuroscience 139(1): 237–49. 16337089 10.1016/j.neuroscience.2005.06.004

[14] Lundqvist M, Herman P, Lansner A (2011) Theta and gamma power increases and alpha/beta power decreases with memory load in an attractor network model. J Cogn Neurosci 23(10): 3008–20. 21452933 10.1162/jocn_a_00029

[15] Nyhus E, Curran T (2010) Functional role of gamma and theta oscillations in episodic memory. Neurosci Biobehav Rev 34(7): 1023–35. 20060015 10.1016/j.neubiorev.2009.12.014 PMC2856712

[16] Palva S, Kulashekhar S, Hämäläinen M, Palva JM (2011) Localization of cortical phase and amplitude dynamics during visual working memory encoding and retention. J Neurosci 31(13): 5013–25. 21451039 10.1523/JNEUROSCI.5592-10.2011 PMC3083635

[17] Uhlhaas PJ, Pipa G, Lima B, Melloni L, Neuenschwander S, et al (2009) Neural synchrony in cortical networks: history, concept and current status. Front Integr Neurosci 3: 17. 19668703 10.3389/neuro.07.017.2009 PMC2723047

[18] Bookheimer S (2002) Functional MRI of language: New approaches to understanding the cortical organization of semantic processing. Annual Review of Neuroscience 25: 151–188. 10.1146/annurev.neuro.25.112701.142946 12052907

[19] Ullman MT (2004) Contributions of memory circuits to language: the declarative/procedural model. Cognition 92(1–2): 231–270. 15037131 10.1016/j.cognition.2003.10.008

[20] Price C (2012) A review and synthesis of the first 20 years of PET and fMRI studies of heard speech, spoken language and reading. Neuroimage 62: 816–47. 22584224 10.1016/j.neuroimage.2012.04.062 PMC3398395

[21] Doesburg SM, Vinette SA, Cheung MJ, Pang EW (2012) Theta-modulated gamma-band synchronization among activated regions during a verb generation task. Front Psychol 3: 195. 22707946 10.3389/fpsyg.2012.00195 PMC3374414

[22] Pang EW, Wang F, Malone M, Kadis DS, Donner EJ (2011) Localization of Broca's area using verb generation tasks in the MEG: Validation against fMRI. Neurosci Lett 490(3): 215–219. 21195135 10.1016/j.neulet.2010.12.055 PMC3076374

[23] Edwards E, Nagarajan SS, Dalal SS, Canolty RT, Kirsch HE, et al (2010) Spatiotemporal imaging of cortical activation during verb generation and picture naming. Neuroimage 50(1): 291–301. 20026224 10.1016/j.neuroimage.2009.12.035 PMC2957470

[24] Korzeniewska A, Franaszczuk PJ, Crainiceanu CM, Kuś R, Crone NE (2012) Dynamics of large-scale cortical interactions at high gamma frequencies during word production: event related causality (ERC) analysis of human electrocorticography (ECoG). Neuroimage 56(4): 2218–37. 10.1016/j.neuroimage.2011.03.030 PMC3105123 21419227

[25] Sirota A, Montgomery S, Fujisawa S, Isomura Y, Zugaro M, et al (2008) Entrainment of neocortical neurons and gamma oscillations by the hippocampal theta rhythm. Neuron 60(4): 683–97. 19038224 10.1016/j.neuron.2008.09.014 PMC2640228

[26] Cheyne DO (2012) MEG studies of sensorimotor rhythms: a review. Exp Neurol 245: 27–39. 22981841 10.1016/j.expneurol.2012.08.030

[27] Kado H, Higuchi M, Shimogawara M, Haruta Y, Adachi Y, et al (1999) Magnetoencephalogram systems developed at KIT. IEEE Trans Appl Supercond 9: 4057–62.

[28] Uehara G, Adachi Y, Kawai J, Shimogawara M, Higuchi M, et al (2003) Multi-channel SQUID systems for biomagnetic measurement. IEICE Trans Electron E86- C: 43–54.

[29] Oostenveld R, Fries P, Maris E, Schoffelen JM (2011) FieldTrip: Open source software for advanced analysis of MEG, EEG, and invasive electrophysiological data. Comput Intell Neurosci 2011: 156869. 21253357 10.1155/2011/156869 PMC3021840

[30] Tallon-Baudry C, Bertrand O, Delpuech C, Pernier J (1996) Stimulus specificity of phase-locked and non-phase-locked 40 Hz visual responses in human. J Neurosci 16(13): 4240–9. 8753885 10.1523/JNEUROSCI.16-13-04240.1996 PMC6579008

[31] Worsley KJ, Marrett S, Neelin P, Vandal AC, Friston KJ, et al (1996) A unified statistical approach for determining significant voxels in images of cerebral activation. Human Brain Mapping 4: 58–73. 20408186 10.1002/(SICI)1097-0193(1996)4:1<58::AID-HBM4>3.0.CO;2-O

[32] Van Veen BD, van Drongelen W, Yuchtman M, Suzuki A (1997) Localization of brain electrical activity via linearly constrained minimum variance spatial filtering. IEEE Trans Biomed Eng 44(9): 867–80. 9282479 10.1109/10.623056

[33] Muthukumaraswamy SD (2013). High-frequency brain activity and muscle artifacts in MEG/EEG: A review and recommendations. Frontiers in Human Neuroscience, 7. 10.3389/fnhum.2013.00138 PMC3625857 23596409

[34] Pfurtscheller G, Lopes da Silva FH (1999) Event-related EEG/MEG synchronization and desynchronization: basic principles. Clin Neurophysiol 110(11): 1842–57. 10576479 10.1016/s1388-2457(99)00141-8

[35] Salmelin R, Hämäläinen M, Kajola M, Hari R (1995) Functional segregation of movement-related rhythmic activity in the human brain. Neuroimage 2(4): 237–43. 9343608 10.1006/nimg.1995.1031

[36] Manto M, Bower JM, Conforto AB, Delgado-García JM, da Guarda SN, et al (2012) Consensus paper: roles of the cerebellum in motor control – the diversity of ideas on cerebellar involvement in movement. Cerebellum 11(2): 457–87. 22161499 10.1007/s12311-011-0331-9 PMC4347949

[37] Burianová H, Grady CL (2007) Common and unique neural activations in autobiographical, episodic, and semantic retrieval. J Cogn Neurosci 19(9): 1520–34. 17714013 10.1162/jocn.2007.19.9.1520

[38] Scoville WB, Milner B (1957) Loss of recent memory after bilateral hippocampal lesions. J Neurol Neurosurg Psychiatry 20(1): 11–21. 13406589 10.1136/jnnp.20.1.11 PMC497229

[39] Jeneson A, Squire LR (2011) Working memory, long-term memory, and medial temporal lobe function. Learn Mem 19(1): 15–25. 22180053 10.1101/lm.024018.111 PMC3246590

[40] Nee DE, Jonides J (2011) Dissociable contributions of prefrontal cortex and the hippocampus to short-term memory: evidence for a 3-state model of memory. Neuroimage 54(2): 1540–8. 20832478 10.1016/j.neuroimage.2010.09.002 PMC2997173

[41] Olson IR, Page K, Moore KS, Chatterjee A, Verfaellie M (2006) Working memory for conjunctions relies on the medial temporal lobe. J Neurosci 26(17): 4596–601. 16641239 10.1523/JNEUROSCI.1923-05.2006 PMC1764465

[42] Öztekin I, Curtis CE, McElree B (2009) The medial temporal lobe and the left inferior prefrontal cortex jointly support interference resolution in verbal working memory. J Cogn Neurosci 21(10): 1967–79. 18855551 10.1162/jocn.2008.21146

[43] Sakai K, Passingham RE (2004) Prefrontal selection and medial temporal lobe reactivation in retrieval of short-term verbal information. Cereb Cortex 14(8): 914–21. 15115738 10.1093/cercor/bhh050

[44] Watson HC, Lee ACH (2013) The Perirhinal Cortex and Recognition Memory Interference. The Journal of Neuroscience 33(9): 4192–200. 23447626 10.1523/JNEUROSCI.2075-12.2013 PMC3736315

[45] Axmacher N, Mormann F, Fernández G, Cohen MX, Elger CE, et al (2007) Sustained neural activity patterns during working memory in the human medial temporal lobe. J Neurosci 27(29): 7807–16. 17634374 10.1523/JNEUROSCI.0962-07.2007 PMC6672876

[46] Burke JF, Zaghloul KA, Jacobs J, Williams RB, Sperling MR, et al (2013) Synchronous and asynchronous theta and gamma activity during episodic memory formation. J Neurosci 33(1): 292–304. 23283342 10.1523/JNEUROSCI.2057-12.2013 PMC3711714

[47] Aminoff EM, Kveraga K, Bar M (2013) The role of the parahippocampal cortex in cognition. Trends Cogn Sci 17(8): 379–90. 23850264 10.1016/j.tics.2013.06.009 PMC3786097

[48] Henke K, Weber B, Kneifel S, Wieser HG, Buck A (1999) Human hippocampus associates information in memory. Proc Natl Acad Sci U S A 96(10): 5884–9. 10318979 10.1073/pnas.96.10.5884 PMC21955

[49] Herrmann CS, Munk MH, Engel AK (2004) Cognitive functions of gamma-band activity: memory match and utilization. Trends Cogn Sci 8(8): 347–55. 15335461 10.1016/j.tics.2004.06.006

[50] Kroes MC, Fernández G (2012) Dynamic neural systems enable adaptive, flexible memories. Neurosci Biobehav Rev 36(7): 1646–66. 22874579 10.1016/j.neubiorev.2012.02.014

[51] Nee DE, Jonides J, Berman MG (2007) Neural mechanisms of proactive interference-resolution. Neuroimage 38(4): 740–51. 17904389 10.1016/j.neuroimage.2007.07.066 PMC2206737

[52] Roy M, Shohamy D, Wager TD (2012) Ventromedial prefrontal-subcortical systems and the generation of affective meaning. Trends Cogn Sci 16(3): 147–56. 22310704 10.1016/j.tics.2012.01.005 PMC3318966

[53] Segal JB, Williams R, Kraut MA, Hart J Jr (2003) Semantic memory deficit with a left thalamic infarct. Neurology 61(2): 252–4. 12874412 10.1212/01.wnl.0000073145.08816.e2

[54] Whitney C, Kirk M, O'Sullivan J, Lambon Ralph MA, Jefferies E (2011) The neural organization of semantic control: TMS evidence for a distributed network in left inferior frontal and posterior middle temporal gyrus. Cereb Cortex 21(5): 1066–75. 20851853 10.1093/cercor/bhq180 PMC3077429

[55] Indefrey P, Levelt WJ (2004) The spatial and temporal signatures of word production components. Cognition 92(1–2): 101–44. 15037128 10.1016/j.cognition.2002.06.001

[56] Goldin-Meadow S, Nusbaum H, Kelly SD, Wagner S (2001) Explaining math: gesturing lightens the load. Psychol Sci 12(6): 516–22. 11760141 10.1111/1467-9280.00395

[57] Marstaller L, Burianová H (2013) Individual differences in the gesture effect on working memory. Psychonomic Bulletin & Review 20: 496–500. 23288659 10.3758/s13423-012-0365-0

